# A Muscle-Driven Spine Model for Predictive Simulations in the Design of Spinal Implants and Lumbar Orthoses

**DOI:** 10.3390/bioengineering12030263

**Published:** 2025-03-06

**Authors:** Robin Remus, Andreas Lipphaus, Marisa Ritter, Marc Neumann, Beate Bender

**Affiliations:** 1 Chair of Product Development, Department of Mechanical Engineering, Ruhr-University Bochum, 44801 Bochum, Germany; 2 Biomechanics Research Group, Department of Mechanical Engineering, Ruhr-University Bochum, 44801 Bochum, Germany; 3 Clinic of Pediatric Surgery, Marien Hospital Witten, Ruhr-University Bochum, 58452 Witten, Germany

**Keywords:** musculoskeletal multibody model, FEM, forward dynamics simulation, lumbar fusion, interbody cage, extensible lumbar belt, soft tissue biomechanics, ArtiSynth, biomedical development process, engineering design

## Abstract

Knowledge of realistic loads is crucial in the engineering design process of medical devices and for assessing their interaction with the spinal system. Depending on the type of modeling, current numerical spine models generally either neglect the active musculature or oversimplify the passive structural function of the spine. However, the internal loading conditions of the spine are complex and greatly influenced by muscle forces. It is often unclear whether the assumptions made provide realistic results. To improve the prediction of realistic loading conditions in both conservative and surgical treatments, we modified a previously validated forward dynamic musculoskeletal model of the intact lumbosacral spine with a muscle-driven approach in three scenarios. These exploratory treatment scenarios included an extensible lumbar orthosis and spinal instrumentations. The latter comprised bisegmental internal spinal fixation, as well as monosegmental lumbar fusion using an expandable interbody cage with supplementary posterior fixation. The biomechanical model responses, including internal loads on spinal instrumentation, influences on adjacent segments, and effects on abdominal soft tissue, correlated closely with available in vivo data. The muscle forces contributing to spinal movement and stabilization were also reliably predicted. This new type of modeling enables the biomechanical study of the interactions between active and passive spinal structures and technical systems. It is, therefore, preferable in the design of medical devices and for more realistically assessing treatment outcomes.

## 1. Introduction

Low back pain is one of the leading causes of disability worldwide and is predicted to increase due to population growth and aging [1]. While its prevalence rises with age, most cases occur between the ages of 50 and 55 [2], resulting in an enormous socio-economic burden, as back pain is a major contributor to absenteeism [1,3,4]. The causes of pain are manifold, and treatment can be complicated when no specific disease or structural cause is known [5,6]. Treatment is generally based on the following two pillars [7,8,9]: conservative treatment, such as psychotherapy, medication, injections, and physical therapy and surgical treatment. In both cases, medical devices are frequently used. However, even when all treatment options have been exhausted, it is often not possible to achieve complete relief of symptoms [9]. A significant proportion of patients often experience recurrent or new problems [10]. To improve treatment for back conditions, reduce surgery and recovery times, minimize post-operative complications, and improve overall quality of life [8,11], new medical devices are constantly being developed [12,13].

Two examples of medical devices used to stabilize the spine are lumbar orthoses for conservative treatment [14,15,16,17] and spinal implants for surgical treatment [18,19]. The latter include screws and rods, interbody cages, and plates that are implanted for the direct fixation and fusion of the spine [20,21,22,23]. A lumbar orthosis, on the other hand, is an external device that encompasses all or part of the lumbar and sacroiliac regions of the trunk and is used to modify the structural and functional characteristics of the neuromuscular and skeletal systems [16,24]. To support the engineering design process [25] and predict the treatment outcomes of new devices, biomechanical studies are conducted [26]. These provide essential quantitative measures to ensure that the design process is not based solely on expert opinion rather than by biomechanical data and clinical evidence [27]. However, even the latest experimental methods used to study spinal mechanics and the loads and effects of medical devices have their limitations [28]. As a result, virtual models are increasingly being used, with varying levels of detail and solution approaches [29,30,31]. These models are intended to describe the existing knowledge of the complex physiology and pathology of the human body using mathematical models in order to derive useful predictions for treatment as a quantitative hypothesis [32]. 

A variety of different virtual spine models have been developed, which can be categorized into the following three types of numerical modeling [29,33]: finite element (FE), musculoskeletal multibody (MB), and combined FE–MB modeling. Each type of modeling has specific advantages and disadvantages and is used to answer different scientific, clinical, and engineering questions. To support the design process of medical devices, FE modeling is most frequently used [30,34]. It enables a detailed structural mechanics analysis of products or engineering designs in interaction with parts of the healthy or pathological osteoligamentous spine [28,35]. To obtain relevant results, realistic boundary conditions must be chosen that reproduce physiologically appropriate spinal loads [36,37,38]. However, the most common limitation of FE models is the absence of the muscles and tissues surrounding the spinal column [34,39]. The effects of muscle forces, body weight, and external loads due to physical activity must be represented by external forces and moments as simplified boundary conditions [38,40,41,42,43,44]. As it is recognized that the loading of the spine is highly complex due to the multiple surrounding redundant intrinsic and extrinsic muscles [45,46,47], the use of simplified boundary conditions can lead to over-simplification and inaccurate predictions of in vivo loading conditions [39,48,49,50,51,52]. Experimental [53] and simulation [54] studies have shown differences in implant loads between simplified and realistic loading conditions, which can be critical if underestimated in the design process. Muscles are also essential for spinal stability [55,56,57,58,59], which is affected by treatment with medical devices such as implants or orthoses [60,61].

To overcome these limitations and retain the advantages of FE modeling, the advantages of musculoskeletal MB modeling can be exploited in the context of a forward dynamic hybrid FE–MB model of the spine [29,33,62,63] with a muscle-driven approach [64,65,66]. These are still rarely used in spinal biomechanics and represent an alternative to coupled FE–MB models [49,52,67,68,69]. A hybrid FE–MB model allows for the calculation of the stresses and strains of specific structures while using rigid bodies and muscles in a single simulation step. This avoids, for example, the technically and mechanically complex coupling of two or more separate FE and MB models, which is necessary for the two-way exchange of simulation results and model synchronization [33,67]. A muscle-driven approach also allows for the simulation of movement and posture by predicting the muscle activation patterns for a dynamic musculoskeletal spine model and its interaction with the environment [66,70]. Such a hybrid FE–MB model can be used, for example, to conduct virtual technical feasibility studies and engineering designs of medical devices to clarify their interactions with biomechanical factors and generate new knowledge to improve patient care [31,71,72,73].

The aim of this study is, therefore, to illustrate a novel model for predicting in vivo-like loading conditions of the lumbar spine treated conservatively or surgically with medical devices. For this purpose, a previously validated intact hybrid FE–MB spine model [74] was modified in three exploratory scenarios, fitted with implants or an orthosis. The model validity was evaluated for different physical activities, including a comparison with data from the literature for the biomechanical responses of the FE and MB components, as well as the internal loads on the spinal implants. As the entire model was muscle-driven, the stabilization mechanisms of the muscle forces were also evaluated and passive minimal models without muscle influence were extracted and validated in advance.

## 2. Materials and Methods

**Figure 1:**
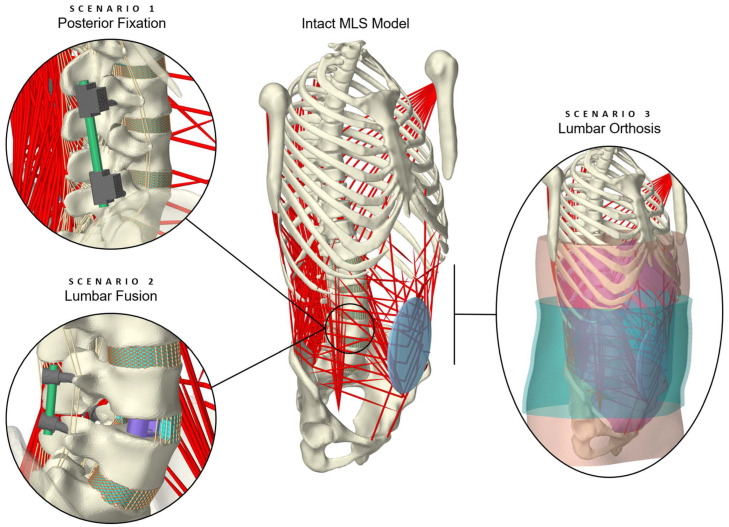
Visualization of the active hybrid FE–MB MLS (musculoskeletal lumbosacral spine) model with scenario overview. The intact MLS model [74] was modified to replicate three treatment scenarios involving medical devices. The procedures can be categorized into surgical treatment in the form of lumbar spinal instrumentation (scenarios 1 and 2) and conservative treatment with an orthosis (scenario 3). For better visibility, the muscles on the right side of the body are hidden in scenarios 1 and 2, and the L4/5 annulus fibrosus is shown in sectional view in scenario 2.

In the following, we describe our approach to modeling three scenarios for the use of medical devices in the lower back and their integration into a musculoskeletal lumbosacral spine (MLS) model for predictive simulations. The inclusion of scenarios was based on available in vivo and in vitro data for validation [27] and consisted of modifications to the intact MLS model (Figure 1). The development and validation of the active intact MLS model, which extended a passive hybrid FE–MB osteoligamentous lumbosacral spine (OLS) model [75], have been described elsewhere [74]. Modeling and simulation were performed within the open-source modeling framework ArtiSynth [76], and the implemented Tracking Controller [77,78] was used to realize the muscle-driven approach for the MLS model. A summary of the details of the intact MLS model relevant to this study is provided in Appendix A.

The predictive simulation of the active hybrid FE–MB MLS model was divided into the following six phases, of which [i], [iii], [iv], and [v] were optional depending on the scenario and physical activity:

[i] First MLS model manipulation during runtime,
[ii] activation of the Tacking Controller and all boundary conditions including gravity, with subsequent 2° flexion and 2° extension of the thorax (spinal settling phase),
[iii] second MLS model manipulation during runtime,
[iv] changing the body posture,
[v] application of external manipulators and loads,
[vi] evaluation of model responses once static equilibrium was reached.

We defined the resulting orientation of the dynamic vertebrae after the spinal settling phase [ii] as the stable, upright reference posture (in the following also abbreviated as standing) with a lumbar lordosis of 52° (COBB angle [79] in between L1 and S1). After reaching a static target posture in [vi], the biomechanical model responses extracted from the MLS model included intradiscal pressure (IDP), intra-abdominal pressure (IAP), range of motion (ROM), intervertebral displacement, and muscle force.

### 2.1. Spinal Instrumentation

Two types of bi- and monosegmental instrumentation were simulated for the first two scenarios. These comprised posteriorly fixed internal spinal fixators spanning from L3 to L5 and L4 to L5, and a L4/5 interbody fusion cage. All implants were implemented by 3D finite elements, geometrically simplified, and symmetrical to the sagittal plane. Their meshes were generated using ANSYS Workbench (2020 R1, ANSYS Inc., Canonsburg, PA, USA) and fed into ArtiSynth. Hexahedral elements were used for the rods. All other elements were meshed with linear tetrahedral elements. Surface nodes of the pedicle screws located within a rigid vertebra were attached to it and the nodes of the screw holes were connected to the adjacent elements of the respective rod in place. All implanted component materials were represented by the titanium alloy Ti6Al4V (Table 1), which was modeled as linear elastic.

**Figure 2:**
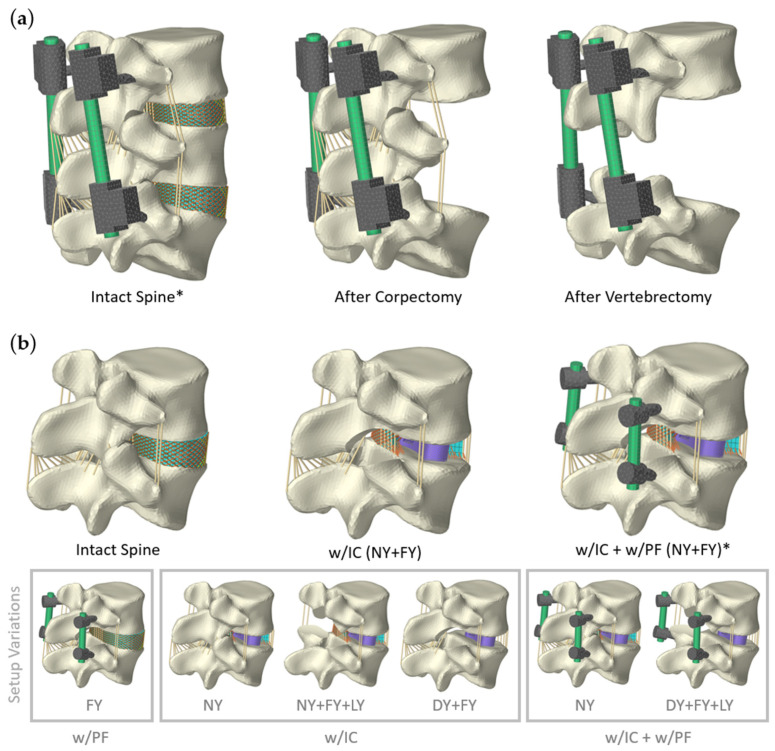
All validation setups for both spinal instrumentation scenarios using sections of the OLS (osteoligamentous lumbosacral spine) model [75]. Only setups marked with an asterisk (*) were used for the active MLS model. (**a**) Instrumented two-level posterior fixators [92] spanning from L3 to L5. Vertebra L4 is bridged in three different clinical scenarios: intact spine, after corpectomy, and after vertebrectomy. (**b**) Implantation of an interbody cage (IC) and bilateral PF (posterior fixation) for single-level spinal fusion. The intact L4/5 functional spinal unit was dissected as shown to replicate a complete bilateral facetectomy (FY) or a FY with laminectomy (LY). The nucleus pulposus was always removed (nucleotomy (NY)) for the IC and the annulus fibrosus remained intact or was entirely removed in a discectomy (DY). For visualization purposes, the annulus fibrosus is always shown in a sagittal section when the IC is implanted.

For preliminary validation of the internal instrumentations, sections of the passive OLS model were used (Figure 2). Moments were applied to the cranial vertebra (L3 or L4) and axial compressive forces with a path optimized follower load [86,87]. The most inferior vertebra L5 was fixed in place. All load cases used are summarized in Table 2.

Subsequently, two instrumented OLS spine sections (*cf*. Figure 2) were transferred unchanged into the MLS model (Figure 1). Muscular structures and adjacent motion segments were not altered. To predict loads on the implants and adjacent level effects [88], sagittal postures were simulated. These were the same as those used to validate the intact MLS model and have been described elsewhere [74].

**Table 2:** Load cases used for OLS model validation with spinal instrumentations. Moments were applied in all spatial directions.

Used for Scenario	Moment in Nm	Follower Load in N	References
1	3.75		[89]
1	6.60		[36,90]
1		250	[36,90]
2	10.0	200	[91]

#### 2.1.1. Scenario 1: Posterior Fixation

The *fixateur interne* described by Dick [92] was utilized to model a bilateral two-level posterior fixation (PF). Rohlmann et al. [36,93] used modified versions [94] of this posterior spinal fixation device to measure the internal loads in the longitudinal rods in vivo and in vitro. We modeled the screws, nuts, and adjustable clamps of the device as a single FE body each. The diameter of pedicle screws was 5 mm and that of longitudinal rods was 7 mm. As a result of the virtual implantation, the maximum unsupported screw length was 12 mm, and the unclamped length of a rod was 40.5 mm.

Von Mises stresses and internal loads on the rods were evaluated in the center between two screw heads. Transformation into an axial force $F_{Z}$ and a bending moment in the sagittal plane $M_{b,sag}$ was carried out according to Rohlmann et al. [36,95]. Due to anatomical conditions, the alignment of the implant coordinate system deviated from that of the upper body. The *y*-axis, which pointed ventrally and ran almost parallel to the pedicle screws, was rotated 15° around the *z*-axis, which represented the longitudinal axis of a rod. Negative values indicated an axial compressive force and a bending moment in flexion.

In a preliminary sensitivity study, we analyzed the influences of different meshes of the PF. When comparing hexahedral elements with linear and quadratic shape functions (Hex8 and Hex20), the changes in internal loads were <0.7% and those in stresses were <4.1%. For quadratic elements in both rods with the final edge length in the longitudinal direction of ≤2.5 mm, the increase in calculation time was, on average, 359% (validation setup after corpectomy, Figure 2a). We ended up using 722 elements for a rod and up to 2890 elements for a single screw clamp combination. The validation of the model was carried out by comparing the internal loads (axial load and bending moment) of the rods with in vitro [36,89,90] and in silico [26] studies under the pure bending moments and pure axial compression forces listed in Table 2. To increase comparability, we modeled three spinal conditions, as shown in Figure 2a.

For predictive simulations with the MLS model, the rotational components of the vertebral target frames were modified. The L4 target frame was removed, and its expected rotational contribution was equally distributed among the remaining vertebral target frames. The expected rotation from L3 to L5 was reduced by 68% (intact OLS with PF compared to no PF during flexion loading), and this reduction was also distributed.

#### 2.1.2. Scenario 2: Lumbar Fusion

Single-level lumbar fusion was modeled, consisting of an interbody fusion cage with supplementary bilateral PF and varying degrees of resection. The diameter of the fixator pedicle screws was 6 mm and that of the straight rods was 5.5 mm. Resections were based on common surgical procedures (e.g., TLIF or PLIF) and are described and visualized in Figure 2b. The L4/5 nucleus pulposus was removed prior to cage insertion. The annulus fibrosus remained intact or was completely removed, depending on the validation setup. In accordance with standard TLIF procedures [19], the cage was banana-shaped, 34 mm wide, with an external cylinder diameter of 82 mm, and had a lordosis angle of 0°. The cage height of 15 mm was determined according to the disc space, thus providing a slight interference fit (press fit).

**Figure 3:**
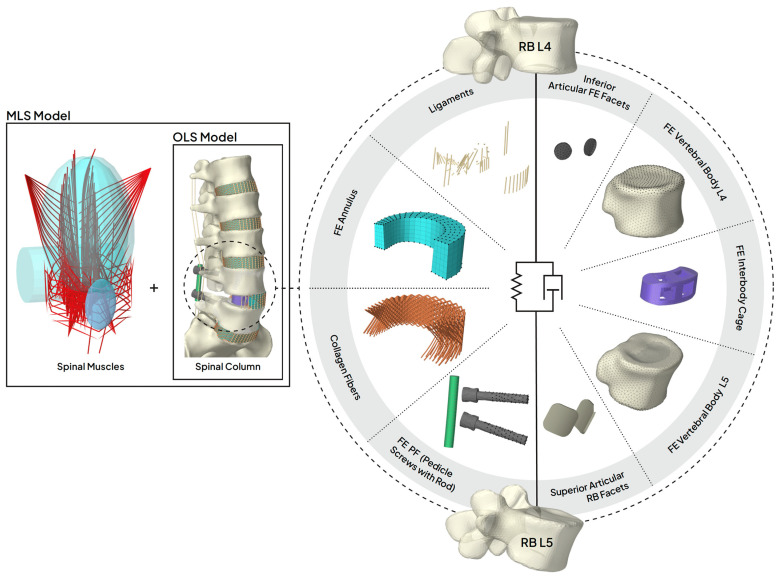
Detailed view of scenario 2 of the MLS (musculoskeletal lumbar spine) model with an exploded view of the L4/5 lumbar fusion. The two subsystems [74,100] spinal muscles and osteoligamentous spine (setup w/IC + w/PF (NY + FY), *cf*. Figure 2b) are shown separately on the left side. All muscles were attached to the RB bones or abdominal plate and were redirected by the cyan colored wrapping bodies in the area of the rib cage and lumbar spine. In contrast to classic musculoskeletal MB models, the mechanical relationship between RB bones were also defined by 3D FE bodies and contact conditions. All the components modeled for this purpose are shown separately on the right (setup w/IC + w/PF (NY)). Using this hybrid FE–MB modeling approach, the dynamic relationships of both RB vertebrae were highly non-linear and modularly adaptable (depending on the setup, components such as facet joints, ligaments, or PF (posterior fixation) were removed or added; *cf*. Figure 2b). All nodes of the FE bodies that were attached to the RB vertebrae are visualized as black dots. These were nodes of pedicle screws, annulus, inferior articular facets, vertebral body L4, and vertebral body L5. The FE cage was placed as a contact body between the two vertebral FE bodies and was only in contact with them. For its caudal contact, the finely meshed endplate of FE vertebral body L5 can be seen. Rods were attached to pedicle screws and start and end points of the ligaments and collagen fibers to RB L4 and L5. Superior articular RB facets were in frictionless contact with the inferior articular FE facets. Note: L4/5 annulus fibrosus is shown in a sagittal section.

For a structural mechanics analysis of the cage and its contacts, the two adjacent vertebral bodies L4 and L5 were integrated into the OLS model as additional 3D FE bodies. The cranial endplate of the FE vertebral body L4 was attached to RB vertebra L4 and the caudal endplate of the FE vertebral body L5 was attached to RB vertebra L5 via their respective nodes. All components of the MLS model and vice versa interacted with these FE models via the RBs L4 and L5. Figure 3 visualizes all the components that defined the mechanical relationship between the RB vertebrae L4 and L5.

**Figure 4:**
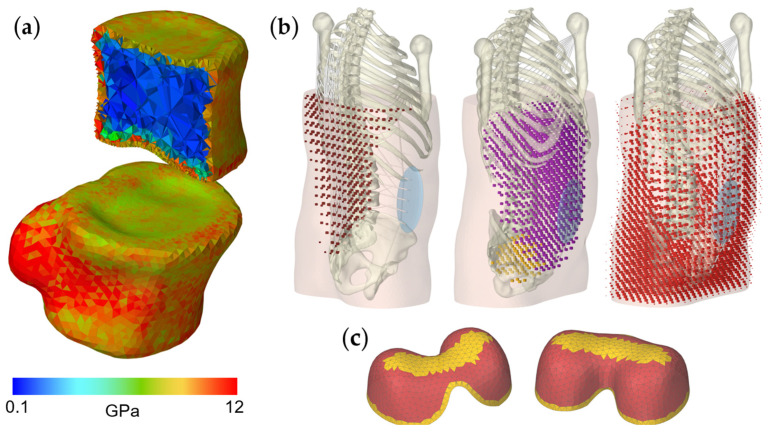
Visualizations of the implemented material heterogeneities. (**a**) Subject-specific Young’s modulus distribution for the FE vertebral bodies L4 and L5 in anterolateral view. Vertebra L4 is cut parallel to the sagittal plane, displaying the internal elements with reduced stiffness. (**b**) Color coding of the FE elements of the embedding mesh to which different material parameters have been assigned: posterior muscle region (left), abdominal and pelvic cavity region (center), and abdominal wall region (right). In all views, the right side of the body is at the front, the left view is posterior-lateral and the central and right view is anterior-lateral. (**c**) Diaphragm shell elements with distinction between muscle (red) and tendon tissue (yellow) in anterior (left) and posterior (right) view.

Linear tetrahedral FE meshes of the vertebral bodies L4 and L5 were automatically generated in ArtiSynth using the TetGen algorithm. The triangular surface meshes used for this were manually segmented from the CT data of the Visible Human Male [96] using 3D Slicer (Version 5.6.2). Assuming a negligible influence for this scenario, the posterior bony elements were removed in the middle of the pedicles. The surface meshes were re-triangulated with SpaceClaim (v201, ANSYS Inc., USA) using the Regularize tool to ensure a sufficient mesh quality. Consistent facet aspect ratios were chosen with maximum edge lengths ranging from 0.5 mm for the end plates to 2.0 mm for the remaining topology (see Figure 3). Automatic mapping of linearly elastic and isotropic material data onto the tetrahedral elements of the vertebral bodies was carried out using BoneMat (Build_152) [97]. Both FE meshes were not symmetrized for this purpose. Each element was assigned an average material property based on the Hounsfield unit (HU) of voxel-wise bone tissue in the CT grid (voxels resampled to 1 mm). Using HU integration with four integration steps and a gap value of 50 for the Young’s modulus calculation and algorithm parameters from Schileo et al. [98] resulted in up to 204 different parameter groups. Since no mechanical tests could be performed to calibrate the material properties [99], the Young’s moduli were scaled to the range from 100 to 12,000 MPa [34,80,81], maintaining their relative distances. Poisson’s ratio was assumed to be 0.3 [80] for all elements. The FE data were imported into ArtiSynth using VTK files along with the material data. The heterogeneous material parameter distributions of both vertebrae are visualized in Figure 4a.

The cage was implanted symmetrically to the sagittal plane in the anterior part of the removed nucleus pulposus. Initially, the cage height was reduced (anisotropic scaling), which allowed for a virtual insertion without contacts and intersections. For subsequent disc space distraction during runtime (step [i]), the cage was first moved caudally to the FE vertebral body L5, attached to it with two central nodes for stability, and then enlarged to its design height. This included physics simulation and contact friction for the cage to be temporarily deactivated. Lastly, the PF was locked and a friction coefficient of 0.5 [101,102] was set for the cage–bone surface contact to simulate the immediate post-operative time. To perform this during runtime, multiple controllers and input probes, which are data streams that can set control inputs and modulate model parameter values over time [103], were implemented.

In a preliminary FE mesh analysis of the rods, the maximum von Mises stresses were convergent with deviations of <5% for maximum element edge lengths of 1.8 mm. The element edge lengths of the screws were 2.5 mm. The element sums for a rod were 456 and were 865 for a screw. When comparing elements with linear and quadratic shape functions for the rods, differences between the results were <0.3% for ROM and < 8.5% for stresses with an increase in the calculation time of 130% (w/IC + w/PF (NY + FY)).

For validation, multidirectional ROMs of the fused L4/5 motion segment were simulated using the same load cases (Table 2) as in the in vitro study by Lund et al. [91]. The following three different conditions of the motion segment were compared: intact, with cage, and with cage and PF (the entire lumbar fusion). The follower load was applied before the pedicle screws were rigidly attached to the RB vertebrae. Different resections (see Figure 2) were simulated to evaluate influences of the OLS model’s biological structures.

For load prediction with the MLS model, the rotational components of the vertebral tracking target frames were modified. The expected rotational contribution from L4 to L5 was assumed to be reduced by 90%, and this proportion was equally distributed among all other vertebral target frames. The physics simulation of vertebra L3 and the Tracking Controller were only activated from step [ii] onwards. Implantation with distraction by cage expansion was performed in the passive state of the MLS model (surgical procedure in prone position, step [i]). In addition to the internal loads of the rods (see Section 2.1.1), contact forces and pressures between the cage and vertebral bodies were also used to evaluate the instrumentation.

### 2.2. Scenario 3: Lumbar Orthosis

**Figure 5:**
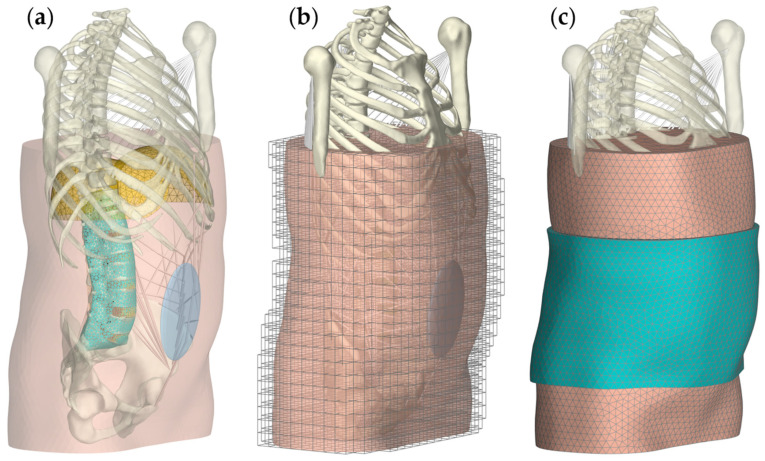
Third scenario, in which the MLS model was extended to include the surrounding FE soft tissue of the trunk and an extensible lumbar orthosis. Three rendering properties are used to visualize different details: (**a**) Inside the transparent torso, one can see the diaphragm (yellow), which was attached to the thorax, and the skinning mesh of the spine (cyan), which was used for the internal contact calculation (spine-soft tissue). (**b**) Surface mesh of the skin that was added to the regular embedding FE mesh as a contact surface. (**c**) Faceting of the polygonal surface mesh of the skin and the FE orthosis. The applied FE orthosis is in its initial state (not tensioned).

In the third scenario, the intact MLS model was extended by a 3D FE model of the surrounding soft tissue of the torso, which was in direct bidirectional interaction with the bony spinal structures. Soft tissue geometries were segmented from the image data of the Visible Human Male [96] from vertebra T9 to the pelvis using 3D Slicer. The flattening of the posterior tissue due to the ventrodorsal weight force [104] was corrected. Within the torso section, the following anatomical structures were separated: diaphragm, thoracic cavity, abdominal cavity, pelvic cavity, back muscles, and abdominal wall (anterolateral and posterior region combined). The diaphragm was derived from the adjacent structures as a surface mesh. All separate geometries were revised in SpaceClaim concerning faceting, sagittal symmetry, and conformity to the MLS model geometry. Additionally, a surface mesh was generated that fully enclosed the lumbar spine except for the spinous processes and that served as a continuous surface for a geometric skinning approach [105,106]. This approach was chosen to obtain a simplified and uniform contact surface that provided a two-way contact force coupling between the soft tissue and the underlying dynamic RB vertebrae. For this, the passive skinning mesh (Figure 5a) was attached to the vertebrae as the master bodies (linear blending), was driven by them, and deformed according to their movements.

In contrast to the common method of meshing free-form bodies with tetrahedral elements, where achieving a high degree of conformity is usually time-consuming and often results in poorly conditioned elements, we used a mesh embedding approach [106,107]. This involved embedding the triangulated surface meshes of the segmented abdominal soft tissue structures in a comparatively coarse but regular grid of 4525 hexahedral elements (Figure 5b). The nodal and element masses and stiffnesses were calculated [107] based on a surface mesh that represented the outer contour of the trunk, excluding the skinned mesh volume of the spine and the thoracic cavity. Two additional surface meshes were embedded for the contact modeling of the skin–orthosis and soft tissue–spine. For the latter, the skinning mesh of the spine was duplicated with an offset of 0.25 mm during runtime at the end of step [ii] to minimize influences on the vertebral dynamics before settling. This contact was assumed to be frictionless [50,108,109] to prevent shear forces between the surfaces.

For an isotropic, hyperelastic, and nearly incompressible material behavior of the abdominal FE elements, the one-term Ogden material model [110,111] was used. The strain energy density function with logarithmic bulk potential is of the form

(1) $$W(\lambda_{i})=\frac{c}{m^{2}}\sum_{i=1}^{3}({\tilde{\lambda }}_{i}^{m}-1)+\frac{\kappa }{2}{\left(lnJ\right)}^{2}$$

where ${\tilde{\lambda }}_{i}$ is the $i^{th}$ deviatoric principal stretch, *c* and *m* are the Ogden material parameters, and *J* represents the volume ratio. Based on the in vivo study by Remus et al. [85], tissue structures were combined and three regions with different material parameter sets were distinguished (Table 1) to obtain a heterogeneous distribution. The element allocations were conducted using the segmented soft tissue geometries and are visualized in Figure 4b. For the posterior muscle and the abdominal wall regions, additional linear dependencies on the excitations of the corresponding muscle groups of the MLS model were incorporated. To do this, the muscle excitations calculated by the Tracking Controller were averaged separately for both regions and the respective material parameter sets (*c* and *m*) were scaled between the minimum and maximum values given in Table 1.

The abdominal cavity was bounded cranially by the dome-shaped diaphragm. This was modeled with 1488 triangle shell elements (Tri3) with a constant thickness of 2.5 mm [112]. All inferior diaphragm boundary nodes were attached to the thorax and three regions [113] for two isotropic linear elastic materials were distinguished (Figure 4c, Table 1), as follows: (1) tendinous region cranial central (phrenic center) and at the border, and (2) the muscular region in between. All other shell nodes were linked to an embedded surface mesh representing the abdominal cavity. Any weight force influences of the FE soft tissues on the MLS model were neglected. To avoid the erroneous application of forces due to boundary conditions, the nodes of the transverse torso sections were fixed caudally to the static pelvis and cranially to an auxiliary body kinematically driven by the dynamic thorax. Further nodes within the bony structures of the thorax and between the ribs were attached to the dynamic thorax. All abdominal nodes within or close to the spinous processes were attached to them. Assuming a rigid pelvic floor, its FE nodes were attached to the pelvis.

The orthosis was an extruded copy of the skin surface facets with a constant height of 24 cm from L1 to the sacrum, assuming a continuous, skintight, extensible lumbar belt (Figure 5c). The resulting 8004 FE wedge elements in two layers of 4 mm each were assigned a linear material behavior (Table 1). The displacement of the caudal orthosis surface nodes was restricted in the vertical body direction. Because measured shear forces between an orthosis and body were low [114], we considered orthosis–skin contact to be frictionless [115].

For a nearly incompressible behavior of the abdomen, the bulk modulus *κ* was set to 40 kPa for all hexahedral elements. This reduced the L3/4 and L4/5 IDPs by about 25% [116] in an upright posture with a maximum pressure of 12 kPa and a mean pressure of 9 kPa under the orthosis. This is consistent with the available literature data, assuming that the orthosis was tightened more, as participants with 8 to 9.4 kPa did voluntarily [60,117,118], in order to simulate a corset inflated to the tolerance limit of the subjects in the study by Nachemson and Morris [116]. To do this, the FE orthosis was tightened during runtime at the beginning of step [v] by continuously reducing its length by up to 8% and temporarily deactivating its physics simulation. The physical simulation of the orthosis was then reactivated, causing it to stretch again, to some extent, due to its elasticity. Contact calculation was permanently enabled.

To evaluate this scenario, we predicted the influences of the soft tissue on the biomechanical responses of the MLS model in different postures. In addition, we simulated the influence of the applied orthosis in an upright posture with and without holding a 20 kg crate in both hands with arms bent in front of the upper body. In the center of the abdominal cavity [109], we examined the IAP via the hydrostatic pressure of the FE elements. We explicitly identify this component as FE–IAP, to distinguish the origin of the IAP for evaluation. Thus, the added FE soft tissue represented a second pressure force source acting cranially on the diaphragm and, hence, the thorax. The IAP resulted from muscle forces acting on the abdominal plate and was not specified in more detail for reasons of consistency. Trunk stiffness was estimated based on the sum of all muscle forces required to maintain a body posture.

## 3. Results

In the following, we distinguish between results using sections of the passive OLS model with simplified loading conditions and results predicted with the muscle-driven MLS model.

### 3.1. Scenario 1: Posterior Fixation

**Figure 6:**
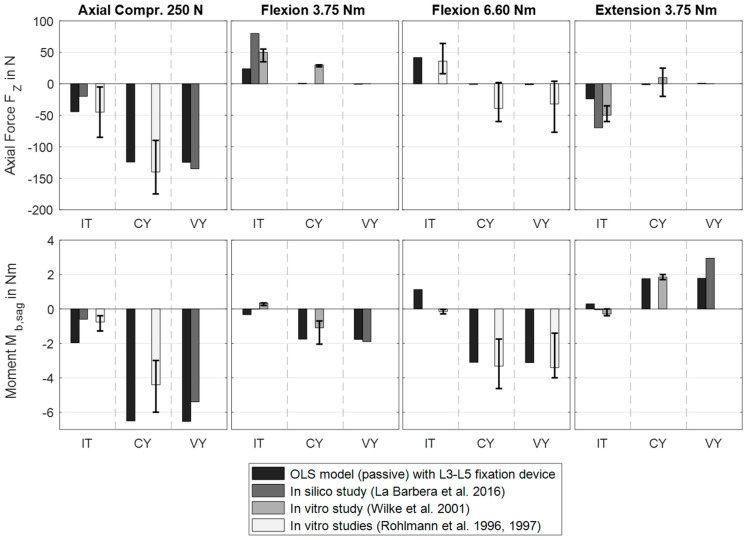
Excerpt from the simulated internal loads in the right rod of the posterior lumbar spinal fixation device (scenario 1) under pure axial compression force or pure bending moment. For validation, the simulation results were compared with in vitro measurements by Rohlmann et al. [36,90] and Wilke et al. [89], and in silico data by La Barbera et al. [26]. Only available comparison data were visualized. The fixators bridged vertebra L4 in three different clinical scenarios (*cf*. Figure 2a): Intact spine (IT), after corpectomy (CY), and after vertebrectomy (VY).

To validate the instrumented internal spinal fixators, the cutting conditions of a rod for the most relevant load cases were compared to data from the literature [26,36,89,90] in Figure 6. The calculated axial forces and bending moments were within the ranges of the published data depending on the spinal condition. Removing biological structures generally increased the load on the rods, which were at their maximum under axial compression. Since our model was not affected by experimental inaccuracies [26], the axial forces in the rods were approximately zero under pure moments after corpectomy. For the intact spine without external loads, $F_{Z}$ was −2.4 N and $M_{b,sag}$ was −0.25 Nm. In the case of corpectomy, both values were reduced to −1.2 N and −0.02 Nm, and in the case of vertebrectomy, to 0 N and 0 Nm.

**Figure 7:**
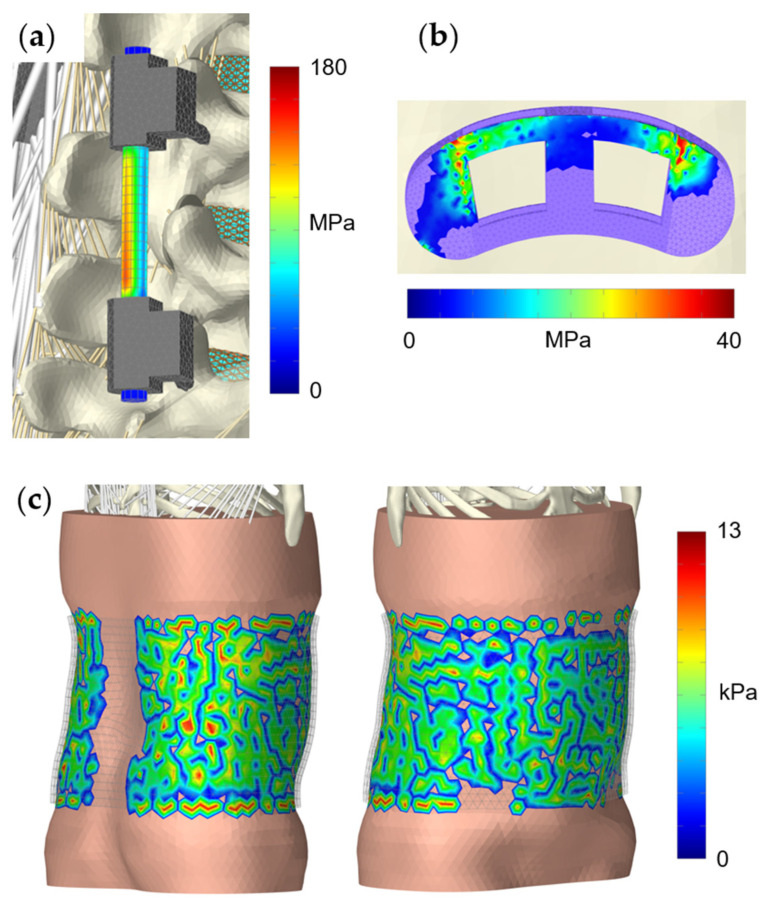
Exemplary visualizations of predicted stress and pressure distributions for the three scenarios: (**a**) Von Mises stress in the right rod of the fixation device in posterolateral view with the thorax in 30° flexion. Muscles on the right side are hidden. The almost stress-free sections of the rod adjacent to the clamps resulted from the attachment conditions of the rod elements. (**b**) Pressure distribution on the cranial side of the interbody cage with the thorax in 30° flexion. All components except the cage were hidden cranial to vertebra L5. (**c**) Pressure distribution under the orthosis applied with maximum tension in relaxed standing position, shown in posterolateral (left) and anterolateral (right) view. The pressure is not interpolated, and the orthosis is displayed transparently.

**Figure 8:**
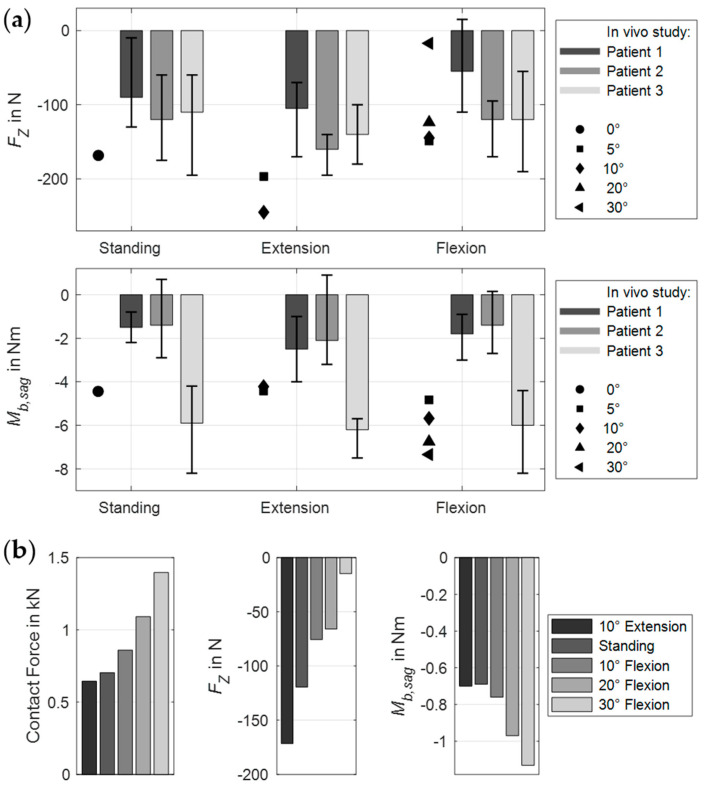
Predicted implant loads in the MLS model for different postures from 10° extension to 30° flexion. (**a**) Two-level posterior fixators implanted in the intact spine (scenario 1, Figure 2a). The predicted axial force components (top) and bending moments (bottom) in the left rod are shown as black symbols for the respective absolute thoracic angle. For comparison, the in vivo data from three patients with anterior fusion from the study by Rohlmann et al. [36] are visualized to the right of each of these. (**b**) For lumbar fusion (scenario 2), the contact forces between vertebral body L4 and the expanded cage (left), as well as the axial force components (center) and the bending moments (right) in the left rod are visualized.

The body postures and resulting muscle activation patterns of the MLS model strongly influenced the internal loads on the PF (Figure 7a). The predicted results compared to in vivo data [36] are visualized in Figure 8a. The forces and moments measured by Rohlmann et al. varied considerably between the three patients. Our results fell almost completely within these ranges and varied depending on different thorax extension and flexion angles. The maximum axial force in a rod was −245 N in 10° extension and the minimum was −12.4 N in 30° flexion. In the latter case, we also measured the maximum bending moment of −7.4 Nm. Thus, the axial forces in extension exceeded the forces measured in vivo. All relative changes due to postural changes were plausible. However, it should be noted that neither the exact postures nor the equivalent spinal conditions of the participants could be replicated in our study.

**Figure 9:**
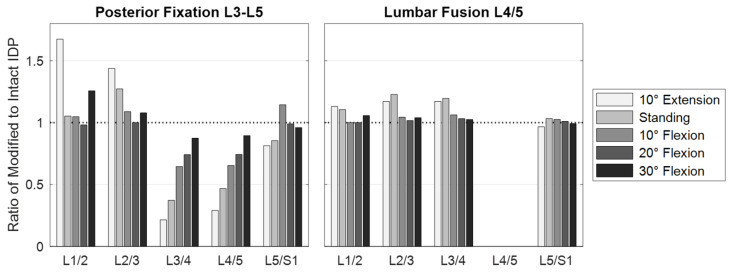
Summary of the predicted IDP changes for the examined postures, in each case as the ratio of instrumented spine to the results of the intact MLS model. The implanted cage replaced the nucleus of the L4/5 disc, which is why no pressure value is available. Refer to Figure 8b for contact forces acting on the cage.

**Figure 10:**
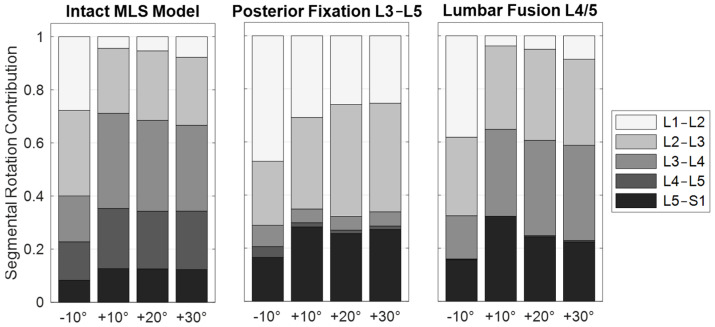
Summary of the segmental rotation contributions of the motion segments L1–L2 to L5–S1 in sagittal plane. The rotation contributions are given for the five postures 10° extension (−10°) to 30° flexion (+30°) for the intact MLS model and the two scenarios with spinal instrumentations. All values are given in relation to the respective upright posture (end of phase [iii]).

**Figure 11:**
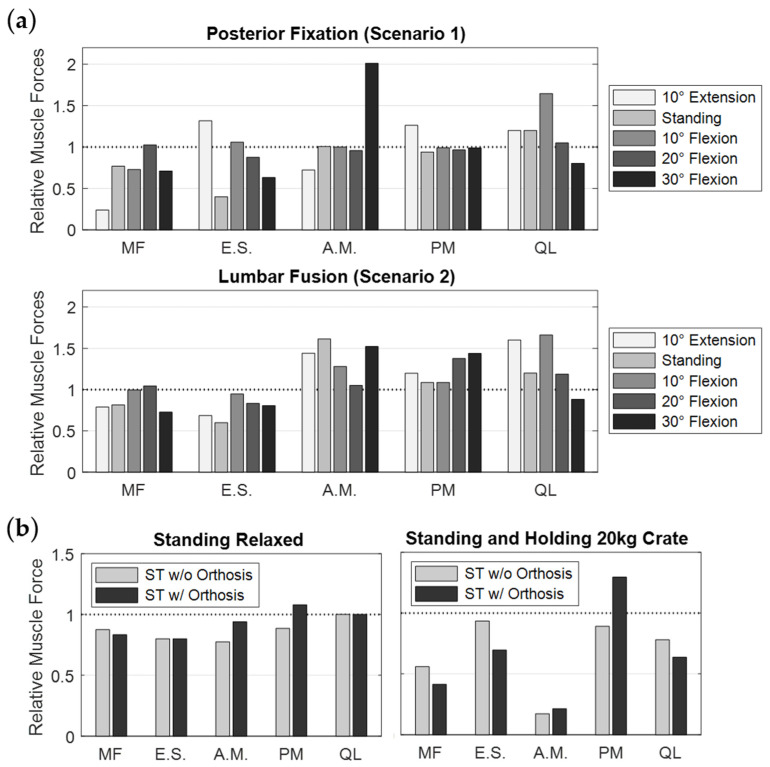
Predicted muscle forces given as the ratio of the modified to the intact MLS model in the same posture. Forces of iliocostalis thoracis, iliocostalis lumborum, and longissimus lumborum are combined into erector spinae (E.S.) and internus abdominis, obliquus externus abdominis, and rectus abdominis are combined into abdominal muscles (A.M.). Further individual muscle fibers were summed for multifidus (MF), psoas major (PM), and quadratus lumborum (QL). Scenarios are the spinal instrumentations (**a**) and the extension with soft tissues (ST) with (w/ orthosis) and without (w/o orthosis) lumbar orthosis (**b**).

Further MLS model responses are visualized in Figure 9, Figure 10 and Figure 11a. The IDP was quantified in the nucleus pulposus of an intervertebral disc as the hydrostatic stress from the node normal stresses. Due to the PF and expected load sharing, the IDPs of levels L3/4 and L4/5 were similarly reduced. The greatest IPD reductions were found in extension (−78% and −71%, respectively). With an increasing flexion angle, the relative pressure differences between unchanged and fixed spinal levels decreased to 13% and 11% (Figure 9). In a previous in vivo study [116], PF resulted in a comparable reduction in disc loading of about 30%. Cranial to the fixed levels, IDPs were increased or unchanged. The pressure increase was maximal in extension and minimal in 20° flexion. For 30° flexion, the IDP change increased again. Except for 10° flexion, the pressure on level L5/S1 was reduced due to PF. The segmental rotation contributions of the lumbar motion segments were altered by the PF (Figure 10). The contributions of L3–L4 and L4–L5 were reduced and compensated by the adjacent levels. The rotation contribution of L2–L3 increased most in flexion. The associated changes in muscle forces (Figure 11a) were heterogeneous for the postures examined. As a result of the increased stiffness of the spine, the total muscle force in the upright posture was reduced by 22%. To generate the maximum flexion angle, more force was applied by the abdominal muscles and less by the posterior muscles. In extension, an inverse behavior was observed. The sum of all predicted muscle forces was, thus, reduced by 18% and 4%, respectively.

### 3.2. Scenario 2: Lumbar Fusion

**Figure 12:**
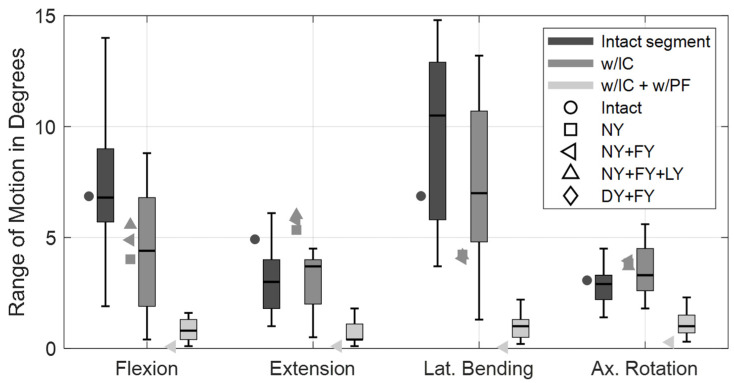
Validation of simulated ROMs of the L4/5 motion segment in intact condition, with interbody cage (w/IC), and with cage and posterior fixation (w/IC + w/PF). The absolute values for the four principal directions are compared with the in vitro data of Lund et al. [91] (shown as boxplots) for the same loading conditions (Table 2). Our simulation data are shown to the left of the corresponding boxplot, and the motion segment condition is color-coded. Further model variations, which are shown in Figure 2b, are illustrated by different marker symbols. No results are visualized for DY + FY without PF, because no stable state was reached, and for variations of the condition w/IC + w/PF (lightest grey), because ROM did not differ.

The calculated ROMs of the validation load cases for the lumbar fusion are visualized in Figure 12 for a comparison with in vitro study data [91]. For flexion and axial rotation, the deviations from their median values were minimal in the intact state. In extension, our motion segment overestimated and in lateral bending it underestimated the in vitro movements. These deviations were absent compared to the in vitro study [119] used to validate the OLS model [75]. As can be seen in Figure 12, these systematic deviations also remained after the implantation of the interbody cage. On the other hand, relative ROM changes with and without the cage were consistent with the in vitro data in all spatial directions. Overall, cage implantation altered the ROMs to between −31% and +21% of the intact movement. The height expansion of the cage increased the disc space by approximately 1 mm, ensured a press fit, and pre-tensioned the structures of the motion segment. This was 6% of the mean disc height, which corresponds closely to previous studies [120,121,122] and was found to mimic the validation results best. The tight annulus fibrosus had the greatest influence on mechanical behavior and stability. For the setups w/IC (NY + FY) and w/IC (DY + FY), distraction resulted in contact forces of 250 N and 120 N on the anterior side of the cage, respectively. Regardless of the validation setup examined, the additional implantation of the PF reduced the ROMs to almost 0° and the stiffness of the lumbar fusion (w/IC + w/PF) was at the upper end of the study data.

In the modified MLS model, the ROM of the fused motion segment L4–L5 decreased to almost 0° in all load cases (Figure 10). While maintaining the total ROM of the thorax, the motion segments adjacent to the lumbar fusion showed the anticipated rotational compensation mechanisms [123]. This is consistent with previous in vivo measurements of spinal kinematics after lumbar fusion [124], in which the loss of intersegmental rotation was compensated by adjacent levels. Proportionally, sagittal intervertebral ROMs were initially compensated caudally and to a greater extent by the cranial segments at larger flexion angles. Overall, the total force in the erector spinae increased with the flexion angle, while extension slightly reduced this force. Except for the posterior muscle groups, the estimated muscle forces were slightly increased due to fusion (Figure 11a). At 30° flexion, these were reduced to 326 N for the erector spinae and to 80 N for the multifidus muscle. The estimated muscle forces were, therefore, lower than in previous in vitro [37] and FE studies [51,125]. Changes in IDPs were calculated to be at least as high or higher cranially from L4/5 (Figure 9). The pressure only decreased in the L5/S1 disc, the most in extension (−4%). This pattern of IDP changes showed a high correlation with the in silico study results of Kumaran et al. [69]. The contact force acting on the cage depended on the postures examined (Figure 7b and Figure 8b). In the upright position, this was 703 N, which agreed with the results of an earlier FE study [126]. The lowest contact force occurred in extension and the highest in 30° flexion. In contrast, the absolute axial forces in the rods were inverted. Maximum axial compression was seen in 10° extension and minimal axial compression in 30° flexion. For the latter, the rod was almost unloaded at −14.8 N. In an upright position, $F_{Z}$ was −119.5 N with a bending moment of −0.7 Nm. In extension and flexion, the bending moment increased to up to −1.13 Nm.

### 3.3. Scenario 3: Lumbar Orthosis

The abdominal FE soft tissue surrounding the spinal column is visualized without (Figure 5a,b) and with (Figure 5c) the orthosis applied. Using the same conditions and physical activities, we found that the soft tissue had an influence on the biomechanical responses of the MLS model. In the upright position, lumbar lordosis was reduced by 1°. The absolute IDP changes were <7.8% (minimal for L4/5 with −2.1% and maximal for L3/4 with +7.8%). In 30° flexion, IDP was +15% at L1/2 and −16.3% at L4/5. The results were within the tolerance range of in vivo studies [127,128,129], but demonstrated the sensitivity of the muscle-driven model to the contact force coupling. We also measured changes in the predicted muscle activation patterns and the resulting muscle forces. The sum of all muscle forces was reduced, as follows: standing relaxed by −1.8%, standing with a 20 kg box by −13%, and in 30° flexion by −38%. While less force was primarily applied by the posterior muscles, the opposite was usually true for the abdominal muscles (Figure 11b). However, since the posterior individual muscle group forces were much greater in comparison, the sums were reduced, and the spinal stiffness was assumed to be increased.

The calculated contact pressure on the skin and the associated soft tissue deformation after the orthosis was fully tightened while standing are shown in Figure 7c. Contact pressure varied with body region and load case. In all cases, the orthosis lifted off the skin in the lumbar region, resulting in zero pressure. This has already been observed in other studies [117,130]. Posterolateral contact pressure was maximal at up to 12 kPa. This resulted in a 23–33% reduction in IDPs and a 0.5° reduction in lumbar lordosis. The predicted FE–IAP increased from 1.1 mmHg to 30.5 mmHg when the orthosis was fully tightened, which slightly exceeded data from the literature [131,132]. The former was the pressure present without orthosis and the IAP remained almost constant at 10 mmHg. The estimated muscle force changes are visualized in Figure 11b. In both cases examined, the posterior muscle groups exerted less force to maintain an upright posture with and without a 20 kg box in both hands. The sum of muscle forces was, thus, reduced by 7.5% and 35% compared to the unmodified MLS model. For these two load cases, the muscle forces with the orthosis applied were reduced by 5.9% and 19.1% for the MLS model with soft tissue (compared to no orthosis). In vivo, trunk muscle activity was similarly little or less affected by elastic lumbar belts under similar conditions [133,134]. The maximum change in muscle force was 81 N for the erector spinae when holding the box. Thoracic rotation differences were negligible. Based on the reduced muscle forces, we assumed that this amount passively increased the stiffness of the trunk and, thus, the spinal stability, without being able to separate the influences of FE–IAP, the orthosis, and other FE soft tissue interactions. Under these simplifying assumptions, the results were in the range of an in vivo study by Cholewicki et al. [15]. In conclusion, all muscles combined contributed less to the active stabilization of the spine against external forces with the orthosis applied. However, the predicted changes in muscle excitation patterns were inconclusive except for the posterior muscle groups.

## 4. Discussion

Determining the internal dynamics of the human spine under realistic loading conditions is an essential step towards a better understanding of the treatment of low back disorders. Therefore, the purpose of our present study was to develop a forward dynamic hybrid FE–MB model capable of predicting the effects of the interplay between passive and active spinal structures, as well as medical devices. The modeling process involved adapting our previously validated active MLS model [74,75] to include different treatment scenarios. The back and abdominal muscles, facet joints, and all spinal ligaments were included. Intervertebral discs, implanted devices, abdominal soft tissue, and an orthosis were modeled using 3D finite elements with linear and non-linear material properties. The comparisons with available in vivo, in vitro, and in silico studies showed a high validity of the three scenarios in the context of the simplified passive OLS and the entire active MLS model.

Even if this model offers a new way to validly study clinical scenarios under enhanced loading conditions, some general limitations must be noted. Firstly, only one model was created and modified in three scenarios. The effects of subject variability could not be addressed. In order to reduce the time currently required for the largely manual creation of such a model, automated pipelines [135] using a deep learning approach [136] may be a way to generate larger cohorts of patient-specific models in the future. Individual segment masses, which are essential for MLS models, could also be more easily predicted from image data using artificial neural networks [137]. Due to the complexity of the model and the scarcity of data from the literature, we validated the model incrementally. This was under the assumption of component validation [138], assuming that models consisting of well-validated sub-models are likely to be valid. The OLS model [75] and the intact MLS model [74] based on it were extensively validated in advance. The investigations in this study were limited to sagittal tasks, the results were evaluated when the entire model reached a static state (equilibrium), and this research was not intended to address specific clinical objectives [16]. However, as a new simulation approach was realized to predict the interdependent effects of implants and orthoses on a muscle-driven musculoskeletal lumbosacral spine model, the three variants were sufficient for a proof of concept. Future studies can include different physical activities to investigate new or patient-specific implant and orthosis designs. Therefore, the proposed model can be used as a basis for a prediction tool for the internal dynamics of the spine in the virtual product design of medical devices. In addition, manufacturers could be supported in proving their compliance with regulatory requirements, necessary under the Medical Devices Regulation (MDR, EU-V 745/2017) before placing such devices on the market in the European Union. These involve demonstrating safety and efficacy, with the potential benefit to the patient increasing with the level of risk.

A key differentiator from comparable spine models is the inherent representation of muscles in the hybrid MLS model. To determine the muscle forces that produced coordinated spinal movements, an optimization routine was utilized. Using a simplifying optimization-based routine, however, may not realistically represent physiological muscle activation patterns lacking a physiological basis, leading to errors in predicted muscle forces [29,139], and internal loads. Validations of the directly correlated spinal loads are, therefore, even more relevant. As appropriate in vivo data are difficult or almost impossible to obtain and are often difficult to justify ethically, the available in vivo studies are limited. For different postures and situations, we compared the results of our model with in vivo data on lumbar IDPs [127,128,129] and internal loads in implanted posterior fixators [36]. There was good to very good agreement, which led us to conclude that the calculated muscle force patterns were valid predictions. It should be noted, however, that in previous studies [140], similar lumbosacral model responses were found for different muscle force predictions for the major spine extensors. As it is very difficult to measure the electrical activity of autochthonous muscles in vivo, there is a lack of data to test mathematical models in this respect [47]. Furthermore, the current optimization routine could not explicitly account for co-contractions of the abdominal and oblique antagonistic muscles [29], as used, for example, when lifting heavy loads [141], which resulted in a tendency to underestimate their activities.

The muscle-driven approach used enabled the forward dynamic MLS model to be moved and held in different body postures without prescribing complete spinal kinematics. However, as partial kinematic specifications were also part of the multi-criteria optimization, an influence is inevitable [142]. For this reason, we examined and minimized the influences of the rotational contributions of the vertebral target frames on the MLS model responses in a previous study [74]. In the first two scenarios of this study, we adopted the hypothesis proposed by Panjabi [123] that a fusion causes an intervertebral motion redistribution that enables the patient to achieve the same ROM as before the surgery. Therefore, we kept the total thoracic ROM and modified the expected rotational contributions of the vertebral tracking target frames based on preliminary examinations with the OLS model. The limitations of this approach were that it is uncertain how individual intervertebral rotation contributions break down and whether patients actually have the same lumbar ROM after fusion surgery. In the in vivo study by Rohlmann et al. [36], for example, patients were asked to move as much as possible without pain. Because the ROM varied, we calculated the internal loads on the fixators for extension and flexion angles from −10° up to +30° and compared the deviations (see Figure 8a). In addition, the pelvis was fixed so that the simulated flexion of the upper body could not represent a combination of hip and lumbar spine movements [125].

Further common limitations of muscle-driven and forward dynamics simulation approaches in the context of clinical biomechanics are a high computational complexity, the required model accuracy, and a *Sim2Real* gap. A predicted movement or posture is the result of a dynamic interplay of all modeled structural components. This requires a high degree of model accuracy concerning all load-bearing structures (*cf*. Figure 3) and the motor control unit, which predicts individual muscle activation patterns [65]. As a consequence, forward dynamics simulations do not generate residual forces and moments, but may continuously diverge from experimental data (e.g., kinematic tracking), which is referred to as *Sim2Real* gap [31]. The computational times were significantly increased with driving muscle excitations compared to the passive OLS model. Using a desktop PC with Intel Core i7-13700K @ 3.40 GHz, 32 GB Ram, and 1 TB SSD running Windows 10 Pro 64-bit, scenarios 1, 2, and 3 took a maximum of 48, 95, and 89 minutes, respectively. Overall, the unavailability of detailed internal loads in other methods emphasizes the importance of a forward dynamics approach as the most powerful approach for investigating changes in mechanical biological spinal structures [143].

As is usual with numerical models [34], this study used an idealized situation for spinal fixation and lumbar fusion. Factors that were disregarded included, for example, pathological bone elasticity, muscle injury, incomplete resection of the cartilage endplate, spinous process fracture, and poor blood supply. All intervertebral discs were modeled as non-degenerated, which is rarely the case for treated in vivo segments [144] and is often the reason for the surgical procedure [37,122]. As a result, both ROMs [145] and IDPs [146] may have been affected. Our internal fixators were simulated without preloading. In reality, this is hardly possible due to the fixation of the screws, results in deviations in the internal loads [89], and can have a strong effect on the mechanical behavior of the bridged region [125,126]. The bone–screw connections were assumed to be ideally bonded, with no relative movement or loosening. Even if this may represent a realistic scenario after complete osseointegration [147], a stiffer construct can be assumed for the primary stability condition tested here. For the posterior fixations and the cage, the available reference data could not be exactly replicated, which may be a factor contributing to differences in the biomechanical model responses. For the screws, this does not allow for a valid assessment of their failure or stress intensifications [147,148]. Our focus was on the rods and their internal loads, which were comparable to study data. The implanted cage was of a different type, narrower, and positioned more anteriorly compared to the cages in the reference study. However, in vitro studies showed that there were no significant differences between cage types in ROM with [149] and without [22,91] PF. Segment stiffness generally increases as the cage size increases, as shown in a numerical study [150]. A further simplification of our current modeling relates to cage insertion and the evaluation of cage subsidence. No bone grafts or fragments were placed in and around the cage, as is usually done [151], which contributes to better osseointegration and fusion [152]. Consequently, it can be assumed that the primary stability was reduced, since the cage contact area was smaller and the stress distribution was less homogeneous (*cf*. Figure 7b).

The interaction between the trunk and an orthosis is highly complex, with a variety of biological and technical factors influencing each other [24]. The modeling in the third scenario was a feasibility study for extending a detailed muscle-driven spine model to include abdominal soft tissue. Although the full validation of such a model is impossible [61], the validity of the underlying components has been confirmed in previous studies [74,75,85]. In contrast to the first two scenarios, no passive OLS model components could be compared with in vitro studies. To the best of our knowledge, there is no comprehensive study of the exact mechanism of action of lumbar orthoses [153] that would allow for the estimated biomechanical model responses to be compared under similar conditions. Only separate biomechanical responses of model components could be quantitatively compared with independent data from the literature, as follows: the IAP resulting from external radial compression [131], the pressure on the skin [60,117,118], the stiffness and deformation of the active abdominal tissue under local compression [85], and the IDP [116]. Although moderate to good correlations were found, this is the main limitation in assessing the validity of the third scenario.

Further limitations and potentials, the influences of which we consider to be particularly relevant for scenario 3, concern the diaphragm, abdominal wall, and IAP. For the diaphragm, its material properties, geometry, and function were simplified. These include orthotropic and muscle-activation-dependent non-linear material properties [82]. Furthermore, it was not taken into account that breathing and body posture influence the morphology of the diaphragm [154] and how this, together with pelvic floor contraction, contributes to an increase in IDP [155]. The abdominal wall also has a decisive influence on the IAP, the biomechanics of which depend on the variable IAP, the passive mechanical properties of fascial and muscle tissue, and the activation of the abdominal muscles [156]. The active abdominal wall is, thus, deformed both by breathing and muscle activity [157,158], as well as by the external radial compression of an orthosis [159]. One way of taking the biomechanics of the abdominal wall into account is to directly embed detailed, active fiber and aponeurosis structures into the abdominal hexahedral mesh [160]. Another option is to use a realistic active 3D FE model where the volumetric abdominal wall is modeled with separate muscle layers and muscle contractility [161]. This active compression of the abdominal content is likely to further increase the stabilizing effect of the trunk already observed in this study. In addition to the IAP, the spine compression force is expected to be increased by the co-contraction of the trunk muscles [158]. The multipoint transversal and oblique abdominal muscles of the MLS model could be replaced and the volumetric muscles could be activated by the Tracking Controller [106]. This has the advantage that the IAP acting on the thorax can only consist of the second component, as follows: (1) a surrogate force calculated from the sum of the abdominal muscle forces acting posteriorly on the abdominal plate (*cf.* Appendix A) and (2) the forces of the abdominal FE soft tissue acting directly on the diaphragm.

Any time-related effects on the abdominal FE soft tissue were neglected. We only examined the immediate effects after the orthosis was applied. The interaction between the soft tissue and the spine was realized using simplified contact conditions (geometric skinning of the spine) and nodal attachments. The results showed that trunk stiffness, quantified by a decrease in predicted muscle force, was increased for the same postures compared to the intact MLS model without soft tissue. This was inevitable with the current approach and must be taken into account when interpreting the results. An additional and expected increase in trunk stiffness occurred due to the external radial compression of the abdomen. The orthosis modeled corresponded to a tall and flexible belt that was tightened more than usual. In this form, it represented a simplified version of orthoses for conservative treatment and is not comparable to weightlifting belts. The latter are narrower, usually made of leather, and, therefore, almost inextensible [162]. Orthoses for treatment purposes are usually higher posteriorly than anteriorly, closed asymmetrically, and made of different fabrics with integrated reinforcements [60,117,153]. This allows for the targeted application of forces to the pelvis, chest, and abdomen, for example to decrease the lordosis angle [163,164] and to posteriorly shift the abdominal center of mass [24]. Studies have shown that the design of the orthosis has an influence on trunk stiffness [60,165], ROM [166], and muscle activity [167]. Consequently, our results were specific to the modeled orthosis in the applied position.

## 5. Conclusions

In this study, we introduced a muscle-driven model of the lumbosacral spine to examine the effects on the musculoskeletal system under realistic loading conditions during conservative and surgical treatment with medical devices. Three scenarios were realized in which the spine was stabilized with implants or an orthosis and different physical activities were simulated. The predicted loading conditions accounted for local and global musculature, body weight, intra-abdominal pressure, and forces resulting from the support of the abdominal soft tissue. The validation of the responses of biological structures and internal loads of implants showed a high degree of agreement with in vivo studies where available. In addition, the model enabled the analysis of structures adjacent to the treated segments for altered spinal loading and load sharing, which may allow hypotheses to be made about adjacent segment disease. Thus, this model represents a new approach to computational spine biomechanics by combining optimization-driven muscles, intervertebral discs, ligaments, facet joints, abdominal soft tissue, implants, and orthoses in one forward dynamic model. In the future, it can be a feasible tool for preliminary clinical evaluation and rapid assessment in the biomedical engineering design of medical devices for conservative and surgical treatment of the lumbar spine.

## Figures and Tables

**Table 1:** Material properties of the anatomical and technical FE components. *c* and *m* are material parameters for the Ogden material model. All other material data of the MLS model can be found elsewhere [74,75].

Component	Material Properties		References
	Young’s Modulus	Poisson’s Ratio	
Bone (cancellous and cortical)	0.1 … 12 GPa	0.30	[34,80,81]
Diaphragm muscle tissue	5.32 MPa	0.33	[82]
Diaphragm tendon tissue	33 MPa	0.33	[82,83]
Implants (Ti6Al4V alloy)	110 GPa	0.30	[80,84]
Lumbar belt fabric	3 MPa	0.49	[50]
	***c***	***m***	
Abdominal and pelvic cavity region	7 kPa	17	[85]
Abdominal wall region	10 … 19 kPa	19 … 22	[85]
Posterior muscle region	25 … 100 kPa	19 … 23	[85]

## Data Availability

The source code of the validated passive hybrid OLS model is openly available at https://github.com/RemusR9/artisynth_lumbosacralSpineModel, accessed on 30 January 2025. Additional data and source code supporting the conclusions of this article will be made available by the authors on request.

## Abbreviations

The following abbreviations are used in this manuscript:

CY	Corpectomy
DY	Discectomy
IC	Interbody cage
IT	Intact spine
FE	Finite element
FY	Facetectomy
HU	Hounsfield unit
IDP	Intradiscal pressure
LY	Laminectomy
OLS	Osteoligamentous lumbosacral spine
MB	Multibody
MLS	Musculoskeletal lumbosacral spine
PF	Posterior fixation
PLIF	Posterior lumbar interbody fusion
ROM	Range of motion
TLIF	Transforaminal lumbar interbody fusion
VY	Vertebrectomy

## Appendix A

The muscle-driven MLS model was created using ArtiSynth (www.artisynth.org, accessed on 30 January 2025), which allowed FE method and musculoskeletal MB dynamics to be combined in one model and solved directly. The modeling details can be described based on the three subsystems that work together in the concept of the spinal stabilization system [57,100]: (1) Passive spinal column, (2) muscles surrounding the spine, and (3) muscle coordination control unit.

(1) The passive OLS model [75] consisted of five rigid lumbar vertebrae, the rigid sacrum, five fiber-reinforced FE intervertebral discs, facet joints, and seven pre-tensioned ligaments. The discs were composed of a quasi-incompressible nucleus pulposus surrounded by the annulus fibrosus with crisscrossed collagen fiber rings. The collagen fibers were embedded in the hyperelastic ground substance and represented by tension-only springs with non-linear material properties. The facet joints were curved with an initial gap width of 0.5 mm and the inferior articular facets had a thin hyperelastic cartilage layer.

(2) The passive hybrid FE-MB OLS model was extended to include 12 muscle groups of the lower trunk, as well as four auxiliary bodies for muscle attachments and wrapping [168]. The muscle groups were modeled with 129 muscle fascicles for each lateral side of the body using a Hill-type muscle model [169] that comprehensively considered the respective lines of action. The IAP was considered as a force acting cranially on the thorax and calculated from the abdominal muscle contractions compressing the abdominal cavity. For this purpose, the abdominal muscles were attached to a kinematically driven abdominal plate that replaced the ligamentous structures of the abdominal wall.

(3) To determine a pattern of muscle excitations that produced a coordinated movement and, thus, solved muscle and spinal redundancy, we used the Tracking Controller [77,78]. This approach made use of *forward dynamics assisted data tracking* [64,170] and enabled the optimization-based prediction of spinal movements and stable postures without prescribing accurate kinematic data. The mathematical model of the MLS fully described how the positions and velocities of model components changed due to applied forces and moments. In contrast to inverse dynamics [65,143,171], the 258 muscle forces that resulted from the musculotendon lengths, velocities, and activations (iteratively determined by the Tracking Controller) actuated or drove the dynamic MLS model and can be referred to as *muscle-driven forward dynamics simulation* [65,66,70,74,139,169,172,173]. As primary objective of the Tracking Controller with chase control as motion tracking setting, target frames were specified for all dynamic and kinematically independent RB skeletal components (thorax and vertebrae L2 to L5). When minimizing the tracking error between tracking target frames and RBs in each time step, 9 of the total 30 degrees of freedom were included. These were all rotations for the thorax and its translations, but not along the longitudinal axis, and for the vertebrae, solely their rotations in the sagittal plane. The nine degrees of freedom of the five target frames were driven by kinematic constraints, and segmental rotational contributions (spinal rhythms) were taken into account [174]. In this study, the excitation group of the lumbar multifidus, which included all fascicles, was divided into five independent excitation groups on each side of the body. This was based on the cranial muscle insertion points (L1 to L5) and allowed for segmental excitation. Details about MLS model calibration and tracking errors can be found in [74].
